# University Students’ Purchase Intention and Willingness to Pay for Carbon-Labeled Food Products: A Purchase Decision-Making Experiment

**DOI:** 10.3390/ijerph17197026

**Published:** 2020-09-25

**Authors:** Rui Zhao, Meng Yang, Jianxiao Liu, Linchuan Yang, Zhikang Bao, Xinyun Ren

**Affiliations:** 1 Southwest Jiaotong University, Chengdu 611756, China; ruizhaoswjtu@hotmail.com (R.Z.); ym2018201259@163.com (M.Y.); rnxnyn@my.swjtu.edu.cn (X.R.); 2 Department of Land Surveying and Geo-Informatics, Hong Kong Polytechnic University, Hong Kong, China; jianxiao.liu@connect.polyu.hk; 3 Department of Real Estate and Construction, University of Hong Kong, Hong Kong, China; u3004700@hku.hk

**Keywords:** carbon-labeled food, purchase intention, willingness to pay, price premium, auction experiment, consumption experiment, consumer behavior, university student, milk

## Abstract

Carbon labeling describes carbon dioxide emissions across food lifecycles, contributing to enhancing consumers’ low-carbon awareness and promoting low-carbon consumption behaviors. In a departure from the existing literature on carbon labeling that heavily relies on interviews or questionnaire surveys, this study forms a hybrid of an auction experiment and a consumption experiment to observe university students’ purchase intention and willingness to pay for a carbon-labeled food product. In this study, students from a university in a city (Chengdu) of China, the largest carbon emitter, are taken as the experimental group, and cow’s milk is selected as the experimental food product. The main findings of this study are summarized as follows: (1) the purchase of carbon-labeled milk products is primarily influenced by price; (2) the willingness to pay for carbon-labeled milk products primarily depends on the premium; and (3) the students are willing to accept a maximum price premium of 3.2%. This study further offers suggestions to promote the formation of China’s carbon product-labeling system and the marketization of carbon-labeled products and consequently facilitate low-carbon consumption in China.

## 1. Introduction

Food is a daily necessity. Its production and consumption undeniably bring about enormous carbon emissions, considerably contributing to anthropogenic climate change [1,2]. Carbon labeling (or carbon emission labeling), which describes carbon dioxide emissions across food lifecycles and provides a sustainability credential, is conducive to enhancing consumers’ low-carbon awareness and promoting low-carbon consumption behaviors. Therefore, it helps to mitigate food’s environmental impacts and improve food sustainability [3,4,5,6]. The first carbon label was put forward by the UK Carbon Trust (a quasi-nongovernmental organization) in 2006 and was presented in the form of numerical value typically based on a lifecycle-based carbon emissions assessment.

Nonetheless, as expected, carbon-labeled (more broadly, eco-labeled, sustainability-labeled) products often involve price premiums. Put another way, products with carbon labels have a higher price than those without such labels, due in part to extra costs of low-carbon certification and technologies. On the other hand, people are generally concerned with and responsive to the carbon emissions problem. They have a positive purchasing attitude towards carbon-labeled products and are thus willing to pay such a premium. Therefore, investigating people’s purchase intention and willingness to pay (WTP) for environmentally friendly products is critical [7,8]. Many previous studies with differing degrees of sophistication and depth have already done so in various contexts [9]. Hartmann and Apaolaza-Ibáñez [10] examined the relationship between psychological benefits, environmental concerns, and consumers’ attitudes and purchase intention for green energy brands. Schuitema and De Groot [11] investigated how products with low environmental impacts influence consumers’ purchase intention. Similarly, Zhao et al. [12,13] identified the factors significantly influencing consumers’ purchase intention for carbon-labeled food products. Echeverría et al. [14] interviewed 774 Chilean supermarket consumers and found that the WTP varies across products. Wong et al. [15] determined the correlates of consumer perceptions of beverage carbon footprints and carbon labels in Hong Kong. Overall, previous studies enrich our understanding of consumers’ behavior toward environmentally friendly products [16,17,18], but many problems remain to be sufficiently investigated.

People’s purchase intention is affected by various factors, such as external stimuli (e.g., labeling information and price), socio-demographic variables (e.g., gender and age), and psychological factors [19,20,21,22,23]. However, the findings of previous studies are exceedingly mixed, even conflicting. For example, Meyer and Liebe [24] identified a strong relationship between income and green consumption behavior, while Zhao et al. [12,13] argued that the two factors had no significant association. Likewise, Colchero et al. [25] further identified an apparent decrease in beverage purchases for the households at the lowest socioeconomic level since the implementation of a beverage tax. However, they also observed no significant impact of the tax implementation on those at the highest socioeconomic level.

China is the most populous country and the second-largest economy throughout the world. It is also the largest carbon emitter. However, no carbon-labeling scheme exists in today’s China. Additionally, university students (or students enrolled in a university) have a keen environmental consciousness and are receptive to new trends and inclined to be involved in sustainable consumption. They constitute a big group of future low-carbon consumers and contribute to the diffusion of environmental-friendly consumption. To this end, in a departure from existing literature, this study does not look at the general population [26] but extensively focuses on a demographic—Chinese university students. It investigates the students’ purchase intention and WTP for carbon-labeled food products and identifies the determinants of their purchase intention.

To examine how consumers respond to a carbon-labeling scheme, the selection of the food product is essential: they should be familiar with the selected product. Milk is a nutrient-rich liquid food produced by mammals (e.g., cows) and daily consumed goods. Its related carbon footprint emerges in the life cycle of input production, dairy production, dairy processing, transportation, marketing, and waste disposal. Among them, input and dairy production (the raw milk stage) account for the largest carbon emissions [27,28]. Given that university students are typically familiar with milk attributes, such as nutritional components and taste [12], this study exclusively focuses on (fluid/liquid) cow’s milk (abbreviated to “milk” hereafter). It first roughly identifies the attributes potentially influencing consumers’ purchase intention through focus group discussions. An auction experiment is then conducted to determine the influence of the attributes identified in focus group discussions. A field consumption experiment is further carried out to verify the auction experiment results and determine students’ WTP in terms of an acceptable range of price premiums.

This study has the following three objectives: (1) investigating the purchase intention and WTP of university students for carbon-labeled food products; (2) identifying the determinants of consumers’ purchase intention for carbon-labeled food products in China (the largest carbon emitter); and (3) providing a new perspective or analytical approach to carbon label studies and supplementing the research field now dominated by interviews and questionnaire surveys. More specifically, existing carbon label-related studies heavily use structured or semi-structured interviews and questionnaire surveys (favored data collection methods in social sciences) to obtain necessary information from respondents (detailed in the ensuing section). Such methods are reasonably useful but are by no means above reproach. They are still subject to many shortcomings (e.g., high time and monetary costs, interview bias, time-lapse considerations, and potential deviation from actual observed human behaviors), some of which may considerably decrease the validity and reliability of the research findings [29]. As such, this study takes a motivational experiment design and conducts behavioral experiments, including an auction experiment and a consumption experiment, to examine the purchase intention and WTP for carbon-labeled food products of students in a Chinese university. This study, which can sufficiently capture actual observed human behaviors, is expected to enrich our understanding of university students’ carbon label-related behaviors and serve as a valuable supplement to the existing literature. Additionally, this study is conducive to developing a carbon-labeled product market, encouraging more residents to participate in low-carbon consumption, and promoting food sustainability. Locally, it provides policy insights into developing a carbon-labeling system in China.

The remainder of this paper is organized as follows: Section 2 provides a brief review of the existing research on consumers’ carbon-label-related behaviors and its methods. Section 3 introduces methods and data sources. Section 4 shows the results of the auction experiment. Section 5 reveals the results of the field consumption experiment. Section 6 discusses the implications drawn from the results. Section 7 concludes the paper and discusses research limitations.

## 2. Literature Review

### 2.1. Consumer Behavior in Carbon-Labeled Products

Purchase intention and WTP for carbon-labeled products have garnered considerable scholarly attention [30,31]. Vanclay et al. [32] used green, yellow, and black labels to indicate low, normal, and high carbon emissions, respectively, of a product. The authors found that green-labeled products witnessed a 4% increase in sales volume over three months, whereas black-labeled ones experienced a 6% decrease, implying that carbon labeling contributes to low-carbon consumption behaviors. Cohen and Vandenbergh [33] and Hornibrook et al. [34] confirmed that carbon labeling increases direct consumer demand for low-carbon products. However, many studies have argued that existing carbon-labeling schemes did not provide sufficient, meaningful information to consumers, which may complicate consumers’ decision making. For example, consumers found that it is difficult, though not impossible, to perceive the carbon emissions value that the label shows [35,36,37].

Prior studies noted that it is essential to improve transparency in carbon-labeling schemes to facilitate better communication with consumers. Feldmann and Hamm [38] revealed that consumers wanted to know more about products, which requires carbon labeling to address the necessity for product information. The authors further found that consumers are willing to pay for products with price premiums that display more information. Hartikainen et al. [39] posited that consumers had comparatively high preferences if carbon labels allowed comparisons. This was verified by Emberger-Klein and Menrad [40] and Feucht and Zander [7], who indicated that a detailed explanation of labeling improvement was effective in stimulating consumers to purchase carbon-labeled products. Consumers used the label to learn about the sources of products and preferred those with a more environmentally friendly production process [41].

Prior studies also focused on whether consumers are indifferent to carbon-labeled products, as the carbon label itself is limited in providing direct individual utility [42,43]. Borin et al. [44] identified consumers’ primary preference for product quality and value rather than labeling environmental information. Guenther et al. [45] argued that investigating consumers’ behavior related to carbon-labeled products is of paramount importance because it helps consumers understand possible behavioral changes in terms of purchase intention and WTP and identify the factors influencing the shift to low-carbon purchasing behaviors.

### 2.2. Method for Investigation of Consumer Behavior in Carbon-Labeled Products

Previous studies devoted to exploring the influencing factors involved in the purchase intention and WTP for carbon-labeled products are mainly empirical and mainly used research methods of the face-to-face interview and online or offline questionnaire [46,47]. Echeverría et al. [14] designed an open questionnaire to investigate Chilean consumers’ WTP for carbon-labeled products. Li et al. [48] collected 1142 questionnaire surveys in Jiangsu Province, China, and developed logistic regression models to identify the main factors that impact consumers’ willingness to buy low-carbon products. Zhao et al. [13] conducted a large-scale questionnaire survey in Chengdu, China, to collect data on consumers’ perception of and purchase intention and WTP for carbon-labeled products. Based on 1132 valid questionnaires, they calibrated logistic regression and ordered logistic regression models to determine the correlates of the three variables concerned. Based on data from a questionnaire survey in Hong Kong, Wong et al. [15] estimated a structural equation model to reveal the association between consumer perceptions of beverage carbon footprints and carbon labels and a series of factors such as gender, age, and education attainment.

Apart from the most commonly used research methods described above, focus group discussions are used in some studies. For example, Upham et al. [30] applied a focus group discussion method to review people’s attitudes toward carbon labeling in grocery products and discussed potential factors that influence their purchasing motivations. Guenther et al. [45] employed focus group surveys to distinguish attitudes toward carbon-labeled products between consumers in the United Kingdom and Japan.

Behavioral experiments have been conducted by a handful of researchers in studying carbon label-related behaviors. Shuai et al. [23] conducted a carbon-labeling scenario experiment in six Chinese cities and concluded that consumers with different socio-demographic characteristics have varying WTP for low-carbon products; and that education level and monthly income are positively related to the WTP. Vecchio and Annunziata [49] carried out an auction experiment in the University of Naples and developed logistic regression models to assess price premiums for food products (more specifically, chocolate bars) with sustainability labels, including fair-trade labels, biodiversity labels, and carbon labels.

### 2.3. Thrust of This Study

The existing literature primarily focuses on either carbon label-related behaviors of the general population or the variances of different groups’ behaviors. It has scarcely looked at a subgroup. Comparatively, this study exclusively centers on university students in China (a big group of future low-carbon consumers in the biggest carbon emitter) and examines their purchase decision-making regarding carbon-labeled and non-carbon-labeled products. It provides theoretical and empirical support to develop low-carbon consumption behaviors.

Methodically, the majority of prior studies used a single experiment to examine carbon label-related behaviors and have seldom integrated different experimental methods. Undeniably, each experiment method has its advantages and disadvantages. The combined use of two or more methods helps maximize strengths and minimize drawbacks, eventually reaching more persuasive conclusions.

## 3. Methods and Data Sources

**Figure 1:**
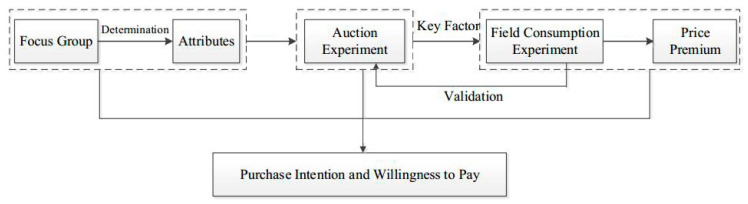
Experiment design of this study.

Figure 1 illustrates the roadmap of this study.

### 3.1. Sample Selection

The experimental group includes 282 students from Southwest Jiaotong University in Chengdu, China, who selected the module “Environmental Protection and Sustainable Development” in the academic year 2015–2016. These students were interested in sustainable development and expressed a keen environmental consciousness. The module was simultaneously offered in four classes in the same academic year. The experimental class was selected using the single-attribute complete randomization, as Table 1 illustrates.

Twenty-four students who chose the class with the smallest random number (curricular number: B3709) were selected to form four focus groups for a semi-structured interview. The semi-structured interview was based on a predetermined set of open questions (see Table 2) and helped obtain the necessary information in a short period [29,50,51,52,53,54,55]. The selection of questions was predominately informed by existing literature on carbon labeling and environmental sustainability (e.g., [13]). The moderator controlled the field procedure during the one-hour seminar and asked the student respondents various questions about carbon-labeled products for their group discussion. Subsequently, the discussion results were applied in subsequent milk auction.

### 3.2. Auction Experiment Design

Students in the class with the largest random number (curricular number: B2133) were chosen as bidders. The entry of the experiment was fully voluntary. Anyone willing to participate was included in the experimental group. Before conducting the experiment, essential requirements were informed, including research aim, duration, procedure, and ethics. The willingness to participate in environmental sustainability research is observed to be rooted in the students’ mindsets. Finally, fifty participants were recruited, as the other ten students exited the auction experiment for personal reasons. The participants were randomly assigned into three groups (numbered as 1, 2, and 3, respectively).

The auction experiment took into account four crucial variables identified in focus group discussions: taste, nutritional components, packaging, and carbon-labeling information. As such, the auctioned products were chosen as (1) 250 mL box-packed pure milk, (2) 243 mL box-packed chocolate milk, (3) 250 mL box-packed high-calcium and low-fat milk, and (4) 240 mL bagged pure milk. Both carbon-labeled and non-carbon-labeled products were auctioned. The information on milk carbon emissions can be found in Zhao et al. [28,35].

As formerly noted, fifty students acted as the bidders (purchasers), while the course lecturer served as the auctioneer. A bidding price range was predetermined, in which milk products’ market prices were used as the price mean and five price levels were set above and below the mean, respectively. Therefore, each type of milk product experienced ten rounds of bidding. In each round of auction, the price increases by 0.1 Chinese Yuan (CNY). Every time the auctioneer quoted a price, the bidders were given seven seconds to decide whether to bid. If they were willing to bid, they could raise their hands. To mitigate the possible interference of social reactions on individual decision making, we asked all participants to respond simultaneously. In such a case, each respondent does not have enough time to interact with others, so the interactive influence can be properly controlled. If a bidder did not participate in a round of bidding, he or she could participate in other rounds. When the bidding prices reached the predetermined ceiling price, the auction was declared as complete.

Multi-round auctions may result in the affiliation effect. In other words, in multi-round auctions, if some bidders discover others’ overbidding, they will follow others to push prices up. Consequently, the final price may deviate from the products’ actual value. Therefore, this study only employed a one-round auction.

### 3.3. Field Experimental Design

**Figure 2:**
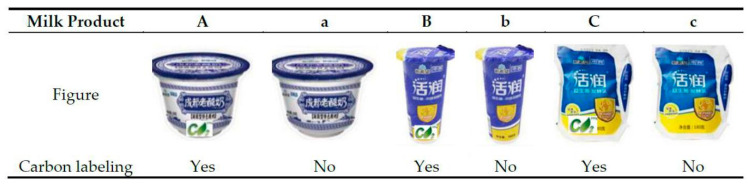
Experimental milk products with and without carbon labels.

This study conducted a field consumption experiment to verify the auction’s experimental results and explore university students’ consumption behaviors influenced by the price differentials between the carbon-labeled and the non-carbon-labeled milk. A small shop on the campus was rented to sell three types of common milk products with the same brand (*huorun*), which differed in price, packaging, and taste. Both carbon-labeled and non-carbon-labeled products were sold. Thus, six samples of milk products were selected, marked as A, a; B, b; and C, c, respectively, as Figure 2 demonstrates.

**Figure 3:**
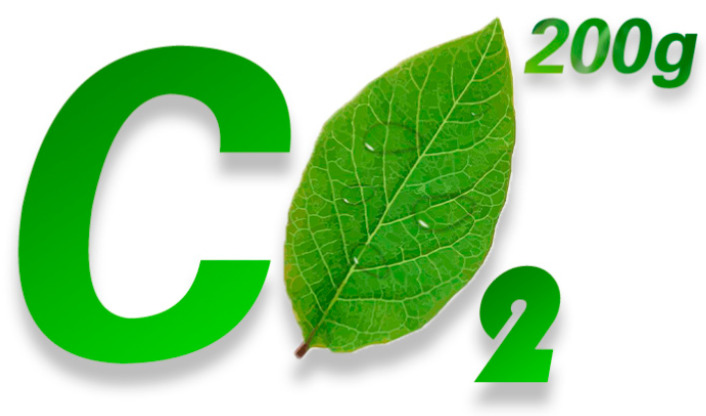
The self-designed carbon label.

However, as China has no carbon-labeling scheme, a self-designed carbon label was marked on the milk package. Our self-designed carbon label has a “CO_2_” shape (Figure 3). The “O” is replaced by a leaf, signifying low carbon, environmental friendliness, and nature. The figure at the upper-right corner of the carbon label indicates the carbon emissions across the product’s lifecycle. This study estimated its emissions to be approximately 200 g for carbon-labeled products, due to the time and cost restrictions on the experimental milk’s carbon emissions throughout its lifecycle, according to previous studies [28,56].

The sale’s target group involved 208 (= 67 + 88 + 60 + 67 − 24 − 50) students in the four experimental classes who did not participate in the focus group discussions and the auction experiment. Sixteen students were unwilling to join, and the other 192 were finally engaged in the selling experiment. Each student joining the experiment received one cash coupon with his or her student ID, which allowed them to purchase one specific type of milk product once. After a student bought a milk product, his or her cash coupon would be collected.

The selling experiment was implemented in three consecutive weeks, and one week corresponds to one period. This study designed three price premium levels based on prior studies on the price premiums of the carbon-labeled milk, as Table 3 notes [12].

## 4. Auction Experiment Results

Data collected in the auction experiment were first analyzed using descriptive statistics and an independent-sample *t*-test in the SPSS 19.0 software (IBM Corporation, Armonk, NY, USA) to investigate whether and how the carbon label impacted the bidders. The experimental data were further subject to a partial correlation analysis to identify the correlation between each control variable and the percentage of bidders.

### 4.1. Descriptive Statistics

**Figure 4:**
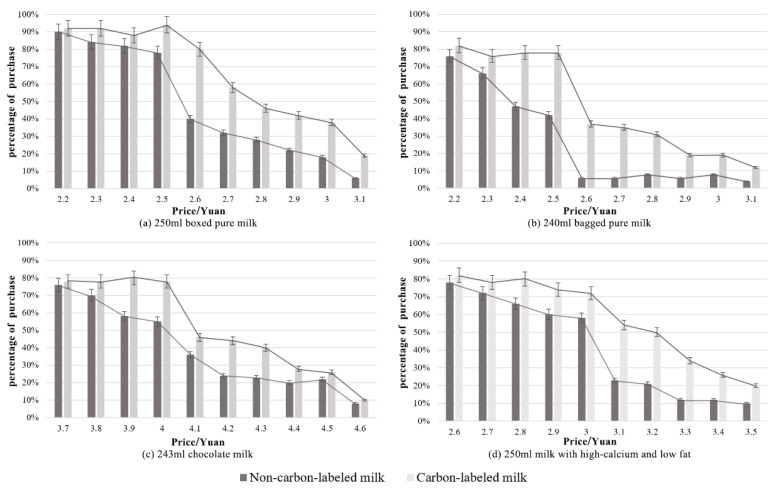
Variations in the percentage of bidders for the four milk products.

Figure 4 presents the variation and indicates the error (or uncertainty) in bidders’ percentage for the four types of milk products, carbon-labeled and non-carbon-labeled. For the same auction price, the percentage of bidders for the carbon-labeled milk is generally higher than that for the non-carbon-labeled milk. With the increase in prices, a decrease in the bidders’ percentage for both the carbon-labeled and the non-carbon-labeled milk is seen, which is highly reasonable. Figure 4 also shows that for the same auction price, the difference in bidders’ percentages for the carbon-labeled and the non-carbon-labeled milk is statistically significant in most cases, although there are still some price levels where error bars overlap. Table 4 shows the maximum differences in the percentage of bidders and their corresponding auction prices.

An independent-sample *t*-test was conducted to verify whether people have different intentions to buy the carbon-labeled and the non-carbon-labeled milk. Its result indicates that the mean percentage of the bidders for the carbon-labeled milk is 16% higher than that for the non-carbon-labeled milk (*t* = 4.306, *p* < 0.001). It also reveals that a carbon-labeling scheme has a positive influence on university students’ bidding behaviors.

### 4.2. Partial Correlation Analysis

Partial correlation analysis, which effectively removes the impact of control variables, is employed to measure the degree of association between a variable (e.g., price, taste, nutritional component, package, and carbon label) and the percentage of bidders. Table 5 reveals the results.

The price is strongly correlated with the percentage of bidders, indicating that price is the core influencing attribute of the university students’ consumption behaviors. This agrees with reality and is consistent with conclusions from prior studies [57]. By contrast, the carbon label modestly affects the percentage of bidders, suggesting that the consumers’ primary concern is the product’s basic attributes [12]. Only when product attributes satisfy their consumption needs will they consider additional attributes, such as the carbon label [39,58].

The milk price is negatively correlated with the percentage of bidders, meaning that a rise in the milk price results in a decrease in bidders, keeping other factors fixed. In addition, carbon labeling, taste, and nutritional components are all positively correlated with the percentage of bidders. More specifically, the carbon-labeling scheme, diversity of milk tastes, and improved nutritional value increased the number of bidders.

## 5. Consumption Experiment Results

Table 6 displays the sales volume of the carbon-labeled and the non-carbon-labeled milk in the three periods. In the first period, no price differential was set between the carbon-labeled and the non-carbon-labeled milk. Overall, 84.6% of the participants purchased the carbon-labeled milk, and the other 15.4% bought the non-carbon-labeled milk. This indicates that university students were more inclined to buy the carbon-labeled milk.

In the second period, during which the price premium increased to 0.1 Yuan, 78.9% of the participants chose to purchase the carbon-labeled milk, and the other 21.1% chose to buy the non-carbon-labeled milk.

In the third period, during which the price premium increased to 0.2 Yuan, 56% of the participants bought the carbon-labeled milk, and the other 44% purchased the non-carbon-labeled milk. The percentage of purchasers of the carbon-labeled milk was not significantly different from that of the non-carbon-labeled milk, which indicates a significant decrease in the university students’ WTP because of an increase in the price premium.

The sales volume of either No. 1 or No. 2 milk is greater than that of the No. 3 milk in each period, indicating that the university students were inclined to purchase the box-packed milk products, keeping the price premium fixed.

**Figure 5:**
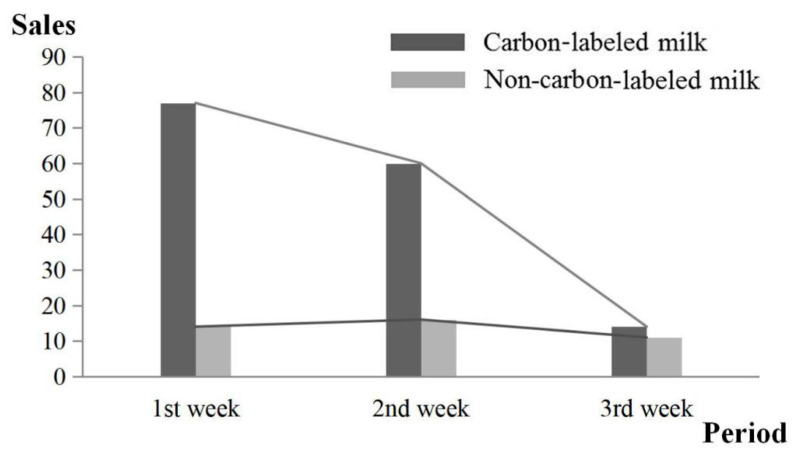
Sales volume of milk in the three periods.

Figure 5 indicates that the sales volume gradually decreased for both the carbon-labeled and the non-carbon-labeled milk with increasing price differential. The sales volume of the carbon-labeled milk decreased more quickly than that of the non-carbon-labeled milk. In other words, the carbon-labeled milk’s sales volume in the first or second period was far greater than that of the non-carbon-labeled milk. In the third period, the volume difference diminishes.

When the price premium increased to 0.1 Yuan, the sales volume of the carbon-labeled milk did not considerably decrease. However, when the price premium increased from 0.1 to 0.2 Yuan, the sales volume of the carbon-labeled milk substantially decreased. This outcome indicates that the price premium of 0.1 Yuan was generally acceptable for university students, who are reasonably concerned with the affordability issue. Given that the average price of the non-carbon-label milk in this experiment was 3.1 yuan, a price premium of 3.2% (=0.1/3.1) seemed appropriate. This phenomenon can be explained by Lombardi et al. [18]: University students are deemed rational consumers with no independent sources of income. As milk is a daily necessity, university students are acquainted with its attributes, including the market price and possible price fluctuations. Suppose the students feel the price premium is too high. In that case, they will be unwilling to buy the carbon-labeled milk, even though they commonly have high educational levels and environmental awareness.

## 6. Discussion

This study sequentially implemented focus group discussions, an auction experiment, and a field consumption experiment to determine factors influencing the purchase of carbon-labeled products (more specifically, milk). The focus group discussions reveal that the purchase of the carbon-labeled milk is mainly influenced by the price, taste, packaging, and nutritional value. The auction experiment and the field consumption experiment further indicate that price was the primary factor influencing consumers’ intention to buy carbon-labeled products. Consumers are unwilling to buy if they feel that the price premium is too high [59,60]. Specifically, university students with no independent financial capabilities are vulnerable to price premiums in choosing carbon-labeled products [14,61].

The consumption experiment indicates the carbon level’s acceptability to consumers (e.g., acceptable maximum price differential). When the price premium was 0.1 Yuan, many students still chose to purchase the carbon-labeled milk. This result implies that most consumers were insensitive to the price premium within a specific price range. Echeverría et al. [14] drew a similar conclusion: Chilean consumers were willing to pay 29% more for milk and 10% more for bread than their respective average prices. Moreover, when the price premium increased from 0.1 Yuan to 0.2 Yuan, an apparent decrease in the carbon-labeled milk sales was witnessed, which suggests that multiple premium points might cause a sudden change in purchase intention. Moreover, the carbon-labeled milk had a higher sales volume than the non-carbon-labeled one, within a premium range acceptable to the experimental group. This indicates that the carbon-labeled products were potentially in demand among the university student group.

We noticed that many students accompanied their roommates, classmates, or friends to our rented shop during the consumption experiment and consulted with their companions regarding the purchase of the milk. Companions are found to be a trusted group of consumers, so their opinions may influence individual consumers’ perceptions of products’ utilities and even cause changes in consumers’ preferences [62,63]. Additionally, companions often share their purchasing experiences, and their positive comments will increase the purchasing confidence of an individual [64]. By contrast, the companions’ indifference to or negative comments about a product will considerably diminish the consumer’s purchase intention [65].

Over the past few years, products or services have been suggested to show their lifecycle-based carbon emissions in the form of carbon labeling to incentivize low-carbon consumption [49]. Though the carbon label intends to raise consumers’ environmental awareness of climate change, it may impose a few challenges on consumers’ decision making. For example, two products equal in quality but mistakenly labeled with different carbon emissions may confuse and even irritate/plague consumers. During the consumption experiment, we observed that several students inquired about the meaning of our carbon-labeling scheme attached to milk products when they hesitate over which experimental milk to purchase. Prior studies also revealed such a dilemma. Upham et al. [30] and Zhao et al. [35] identified that the current carbon-labeling scheme was intricate for consumers to perceive a specific value of carbon dioxide emissions and its environmental impact. In this case, enhancing the transparency of the current carbon labeling system may be a useful way to reinforce its environmental communication.

This study is by no means above reproach. Instead, it still has some limitations. First, the key factors influencing consumers’ purchase intention and WTP of university students are identified only from the perspectives of external stimuli or products’ attributes [66,67]. Therefore, socio-demographic and psychographic variables (e.g., gender, age, and habits) are overlooked. Second, we exclusively focus on university students, and the experiment’s sample size is limited. This feature modestly weakens the generalizability of this study. Meanwhile, due to the lack of time and monetary resources, we only sampled a subgroup of university students, namely those who selected the module “Environmental Protection and Sustainable Development.” The sampled students may be more inclined to be supportive of environmental issues and have different environmental attitudes from the rest of the student population. This study, therefore, may overestimate the WTP for carbon-labeled milk of university students. That is, the true premiums for the population of university students may be lower than the 3.2% premium found in our study.

Future studies can proceed from the following aspects. First, they should focus on the discrimination of impacts from internal and external factors by using more rigorous, sophisticated psychologic experiments and should investigate their synergetic influences on consumers’ behavior. More scientific research methodologies (e.g., sampling and survey methods) are also needed.

Second, this study proposes an easily applied analytical framework for the studied problem, but it only looks at a subgroup of the population, namely university students. Evidently, understanding and evaluating the purchase intention and WTP for carbon labeled products is also a timely and important research topic. As such, shifting the focus from university students to the general population is also needed. In addition to the newly proposed analytical framework, this study still has some implications for the estimation of the WTP for the general population. As income is a key factor in positively affecting the purchase of carbon-labeled products [17,23], the premiums for the general population can be reasonably expected to be higher than our estimates (3.2%). Future rigorous studies are expected for the accurate WTP estimation.

Third, this study only uses the label of “CO_2_”. However, enteric fermentation (CH_4_) is a significant contributor to emissions. Using the concept of “CO_2_e (equivalent)”, which can include other emissions such as N_2_O from microbial denitrification of chemical nitrogen fertilizers (which is pertinent in this case given corn fed to dairy cattle), is needed because the new label (“CO_2_e”) can encourage survey respondents to investigate this nuance and detail. Future surveys should do so rather than using the “CO_2_” label.

Last, assessing the own-price elasticity of demand (a measure of the responsiveness of the quantity demanded to a percentage change in the price) for these types of milk products for university students (more broadly, Chinese consumers) is essential as it helps to accurately estimate the potential welfare loss (for example, loss in consumer surplus) for consumers who drop out of the potential market for these types of milk products. As Table 6 and Figure 5 show, there was a substantial exit from the market at higher price premiums (especially the third one). If milk demand for Chinese university students is inelastic relative to milk supply, then economic theory suggests that the consumers would bear more loss of consumer surplus compared to milk producers’ loss of producer surplus. By contrast, if the milk demand is more elastic than supply, milk producers would bear more welfare loss. Therefore, we suggest that more studies be devoted to evaluating the own-price elasticity of demand for milk by Chinese university students, which contributes to delving deeper into the neoclassic economic issue of social welfare loss.

## 7. Conclusions

This study employed an auction experiment and a field consumption experiment to observe university students’ purchase intention and WTP for carbon-labeled milk products, in which critical external influencing attributes were identified by focus group discussions in advance. The auction experiment revealed that price is a pivotal attribute to influence consumption behaviors, while the consumption experiment further investigated students’ purchase intention and WTP. The main findings indicate that the milk price is negatively correlated with the bidders’ percentage. However, carbon-labeling information, taste, and nutritional components are positively connected to the percentage of the bidders. The experimental students’ purchase intention for the carbon-labeled milk is modestly correlated with the information disclosed on carbon labels. The university students would accept a maximum price premium of 3.2% toward the carbon-labeled milk.

This study contributes to better understanding consumers’ behavior regarding carbon-labeled products by investigating possible responses from a homogeneous consumer group (university students), thereby providing empirical evidence of low-carbon consumption. As China has not launched a carbon-labeling scheme, this study identifies the attributes that influence the development of the carbon-labeled product (e.g., food) market and promote sustainable development. The methods applied in this study fill gaps in capturing actual consumer behaviors in response to various carbon-labeled product attributes based on hybrid experiment design. Such an experiment helps us to better interpret consumers’ purchase intention and WTP and encourage low-carbon consumption behavior.

## Figures and Tables

**Table 1:** Results of the single-attribute complete randomization.

Curricular Number	Number of Students	Random Number
B2131	67	0.606
B2132	88	0.896
B2133	60	0.976
B3709	67	0.062

**Table 2:** Question design for the focus group.

Number	Question
1	Have you heard of carbon labeling?
2	What do you think of the utility of carbon labeling?
3	Would you buy milk for your daily consumption?
4	Why would you choose to purchase milk?
5	What concerns you when purchasing milk?
6	Would you choose to purchase the carbon-labeled milk?
7	Why would you choose to purchase the carbon-labeled milk?
8	Are you concerned with the labeling information when purchasing milk?
9	Do you think the carbon-labeled milk will be more expensive?
10	Would you buy the carbon-labeled milk if its price were higher than that of the non-carbon-labeled milk?
11	If so, to what extent would you accept an increased price?

**Table 3:** Price premiums in different sales periods.

Period	Week	Price Premium/Differential (Per Product)
1	1st week	0 Yuan
2	2nd week	0.1 Yuan
3	3rd week	0.2 Yuan

**Table 4:** Maximum and minimum differences in the percentage of bidders and their corresponding prices.

Milk Product	Maximum Difference	Percentage for the Carbon-Labeled Milk	Percentage for the Non-Carbon-Labeled Milk	Corresponding Auction Price
250 mL box-packed pure milk	40% *	80%	40%	2.6 Yuan
240 mL bagged pure milk	36% *	78%	42%	2.5 Yuan
243 mL box-packed chocolate milk	23% *	78%	55%	4.0 Yuan
250 mL box-packed high-calcium and low-fat milk	32% *	54%	22%	3.1 Yuan

Note: * denotes that the difference is significant at the 5% level, which is indicated by two-sample two-tailed *t*-tests.

**Table 5:** Results of partial correlation analysis.

Variable	Partial Correlation Coefficient
Price	−0.876
Taste	0.867
Nutritional components	0.684
Package	−0.592
Carbon Label	0.513

**Table 6:** Sales volume of different types of milk in the field consumption experiment.

Period	Price Differential	No. 1			No. 2			No. 3		
		A (CL)	a (NCL)	Total	B (CL)	b (NCL)	Total	C (CL)	c (NCL)	Total
1	0 Yuan	30	8	38	28	4	32	19	2	21
2	0.1 Yuan	21	7	28	22	5	27	17	4	21
3	0.2 Yuan	5	4	9	6	4	10	3	3	6

Note: CL = carbon-labeled, NCL = non-carbon-labeled.
